# Fish Oil Supplementation as an Omega-3 Fatty Acid Source during Gestation: Effects on the Performance of Awassi Ewes and Their Offspring

**DOI:** 10.3390/ani13243888

**Published:** 2023-12-18

**Authors:** Mustafa M. Alshdaifat, Ugur Serbester, Belal S. Obeidat, Murat Gorgulu

**Affiliations:** 1 Al-Khanasiri Department for Livestock and Rangeland Research, National Agricultural Research Center (NARC), P.O. Box 639, Baqa’a 19381, Jordan; m.shdaifat78@yahoo.com; 2 Department of Animal Science, Agriculture Faculty, Cukurova University, Adana P.O. Box 01330, Türkiye; 3 Department of Animal Production, Faculty of Agriculture, Jordan University of Science and Technology, P.O. Box 3030, Irbid 22110, Jordan; bobeidat@just.edu.jo; 4 Makrovit Animal Feeding Technologies, Konya P.O. Box 10757, Türkiye

**Keywords:** ewes, fish oil, maternal feeding, PUFAs, lamb

## Abstract

**Simple Summary:**

Pregnancy is a critical period in the life cycle of mammals, and proper nutrition plays a pivotal role in the development and health of the offspring. In recent years, there has been a growing interest in the potential benefits of supplementing maternal diets with fish oil, rich in omega-3 fatty acids, during pregnancy. This paper explores the effects of incorporating fish oil into the diet of pregnant ewes on the dams and the development and well-being of lambs.

**Abstract:**

The primary aim of this research was to assess the impact of supplementing fish oil into the diet of pregnant Awassi ewes on various aspects of the dam’s productive performance, offspring birth weight, colostrum yield and quality, milk production and composition, postpartum offspring performance, and the composition of fatty acids in milk. In this study, two different fat sources, namely palm oil (PO) and fish oil (FO), were utilized, with both being included at a rate of 2.4% of dietary dry matter (DM) during the initial 65 days of gestation (early gestation stage), and then at 2.1% DM from day 65 of gestation until lambing (late gestation stage). The study subjects were Awassi ewes with a body weight (BW) averaging 57 ± 1.5 kg and an age of 3.5 ± 1.2 years. These ewes were randomly assigned to one of four dietary treatments: (i) a diet containing only palm oil from day 0 to day 150 of pregnancy (PO, n = 15); (ii) a diet containing palm oil from day 0 to day 65 of pregnancy, followed by a diet containing fish oil from day 66 to day 150 of pregnancy (POFO, n = 16); (iii) a diet containing fish oil from day 0 to day 65 of pregnancy, followed by a diet containing palm oil from day 66 to day 150 of pregnancy (FOPO, n = 16); (iv) a diet containing only fish oil from day 0 to day 150 of pregnancy (FO, n = 16). Within each treatment, ewes were housed in three replicates, with each replicate further divided into three subgroups. The first two subgroups each contained eight ewes, while the third subgroup contained nine ewes. In addition, each treatment contained 10 primiparous and 15 multiparous ewes. The results indicated that the POFO treatment led to significant increases (*p* < 0.05) in birth weight, gestation length, and colostrum IgG concentration. However, it also resulted in a decrease (*p* < 0.001) in colostrum production and a reduction (*p* < 0.001) in the percentage of milk fat and milk protein. Furthermore, the milk from the POFO treatment exhibited higher levels of polyunsaturated fatty acids (PUFAs) (*p* < 0.001) compared to the PO and FOPO treatments, while the milk saturated fatty acids (SFA) were lower (*p* < 0.001). In conclusion, the addition of fish oil at a rate of 2.1% DM during the late pregnancy period showed promise for enhancing birth weight, colostrum IgG concentration, and the PUFA content in the milk.

## 1. Introduction

Ensuring an adequate supply of nutrients during gestation holds significant importance as it supports various crucial aspects, including the growth of fetal organs, colostrum and milk production, maintenance of physiological needs, and development of the mammary gland [1,2]. Multiple studies have demonstrated that omega-3 polyunsaturated fatty acids (n-3 polyunsaturated fatty acids or PUFAs), such as eicosapentaenoic acid (EPA) and docosahexaenoic acid (DHA), are subject to limited or negligible biohydrogenation in the rumen [3]. These fatty acids, which are considered non-essential because they can be synthesized from linolenic acid (n-3), are primarily derived from sources like fish and marine products, including fish oil and fishmeal. Moreover, supplementing with polyunsaturated fatty acids (PUFAs) plays a role in various aspects of reproductive processes, including the establishment of pregnancy, uterine endocrinology, and the prevention of preterm birth [3].

A significant economic challenge in the sheep industry stems from the high rates of fetal and embryonic mortality during pregnancy. This can represent as much as 43% of the flock during the initial 25 days of pregnancy, resulting in approximately 28% of embryonic loss [4]. Supplementation with n-3 PUFAs has been shown to reduce the synthesis of PGF2α and prevent the regression of the corpus luteum (CL), leading to sustained secretion of progesterone (P4). This, in turn, may improve embryo survival and reduce PGF2α secretion [5,6]. It has also been shown that linseed-rich diets with a high n-3 content have the potential to decrease embryo mortality in dairy cows [7]. The inclusion of n-3 PUFAs in diets also influences gestation length and the timing of parturition by altering the quantity and type of prostaglandin (PG) synthesis, given that PGs are essential for parturition [8,9]. This can result in a reduced incidence of premature labor [10]. Ewes fed fish oil or algal DHA during the third trimester of gestation had a gestation length that was two days longer than ewes fed protected fat [11,12]. In addition, [13] found that ewes supplemented with diets containing n-3 polyunsaturated fatty acids from fish oil had a longer gestation length than ewes supplemented with diets containing n-6 fatty acids.

Ruminant rations often face limitations in long-chain fatty acids (FAs), and rumen biohydrogenation is known to be extensive and highly efficient [14]. This can affect the availability of FAs in biological fluids (such as milk or blood), meat, and fetal tissues particularly during the late gestation period when rapid fetal growth occurs. Hence, augmenting the concentration of omega-3 family DHA and EPA in colostrum, milk, and milk products by incorporating them into the diet could be beneficial for suckling animals. On the other hand, biobased molecules released by the Maillard reaction that occurs with the heating during the production of fish oil may possess excellent antioxidant (including metal chelation and free radical scavenging) and emulsifying properties [15,16]. In addition, limited data exist regarding the feeding of diets containing fish oil to small ruminants. Consequently, the objectives of this study were to assess the impacts of feeding fish oil during various stages of pregnancy on maternal performance, embryo survival, morbidity and mortality of newborns, colostrum and milk production, milk FA profile, newborn performance during the preweaning period, and lamb performance during the fattening period in Awassi ewes during pregnancy, as well as its effects on their offspring.

## 2. Materials and Methods

The care of all animals in this study adhered to the respective national guidelines on animal care in Jordan and Turkey.

### 2.1. Study Area

The study was conducted at the Al-Khanasry Station for Livestock Research, which is affiliated with the National Centre for Agricultural Research and Extension (NCARE) in Jordan. This research facility is located approximately 50 km north of Amman, situated at coordinates 32°04′42.4″ N 35°50′32.8″ E and an altitude of 350 m. The station experiences an arid to semi-arid climate, with an annual precipitation of 170 mm.

### 2.2. Animal and Housing

A total of 100 mature Awassi ewes, aged 2–4 years, with parity ranging from 1 to 3 and a body weight (BW) of 57 ± 1.5 kg, underwent a health and udder assessment by a veterinarian prior to the commencement of the study. To synchronize the ewes, intravaginal sponges containing 60 mg of progesterone acetate (Veramix, Pharmacia, and Upjohn Co., Orangeville, ON, Canada) were used for a period of 14 days. After the removal of the sponges, all ewes were randomly divided into 10 equal groups, each comprising 10 ewes. Natural mating took place, with each group assigned a fertile ram at a ratio of 1 ram to 10 ewes. Pregnancy diagnosis occurred during the tenth week of pregnancy, and non-pregnant ewes were excluded. The ewes were accommodated in an environment with natural lighting and ambient temperature conditions. The gestation period was divided into two periods: from breeding to day 65 and from day 66 to lambing. Starting from day 0 of mating, ewes were randomly allocated to one of four dietary treatments: (i) diet containing palm oil from day 0 to day 150 of pregnancy (PO; n = 15); (ii) diet containing palm oil from day 0 to day 65 of pregnancy, followed by a diet containing fish oil from day 66 to day 150 of pregnancy (POFO; n = 16); (iii) diet containing fish oil from day 0 to day 65 of pregnancy, followed by a diet containing palm oil from day 66 to day 150 of pregnancy (FOPO; n = 16); (iv) diet containing fish oil from day 0 to day 150 of pregnancy (FO; n = 16). Ewes were grouped into three replicates per treatment, with each treatment further divided into three subgroups, and they were housed in shaded pens measuring 12 m × 6 m. Each ewe had approximately 33 cm of space at the feeder. After birth, ewes and their lambs were housed in shaded pens, with three replicates per treatment, and all pens were equipped with an area for creep feeding. At the end of the nursing period, lambs were separated from their dams and continued to be grouped with the same ewes during the milking period. The study encompassed 8 months, covering 5 months of pregnancy, 3 months of nursing, and a milking period.

### 2.3. Experimental Diets

The diets were isonitrogenous and provided as a total mixed ration (TMR) to meet the gestation requirements of Awassi ewes (Table 1). Ewes were fed twice daily at 8:00 h and 15:00 h. The fat sources used were palm oil (PO) and fish oil (FO), incorporated at 2.4% of dietary dry matter (DM) during the first 65 days of gestation (early stage) and 2.1% DM from day 65 of gestation until lambing (late stage). The fatty acid profiles of the PO and FO are detailed in Table 2. The roughage-to-concentrate ratio was 45:55 throughout the study. Ewes had ad libitum access to feed and fresh water. Post-lambing, ewes were fed a diet without FO or PO to meet the requirements for nursing [17]. The diets were offered as a TMR, consisting of 50% barley grain, 15% soybean meal, 33% wheat straw, 1% salt, 0.9% limestone, and 0.1% mineral vitamin premix. The diets provided 90.7%, 89.7%, 14.5%, 37.4%, 19.9%, and 2.5 Mcal/kg DM for organic matter (OM), crude protein (CP), neutral detergent fiber (NDF), acid detergent fiber (ADF), and metabolizable energy (ME), respectively. Diet samples were collected in triplicate for laboratory analysis to ensure consistent chemical composition.

Ewes and their lambs were grouped and fed their diets daily at 8:00 h. The animals were housed in 12 pens measuring 12 m × 6 m.

### 2.4. Data Collection and Measurements

Monthly weighing of ewes occurred before the morning feeding throughout the study. Nutrient intakes were assessed by collecting orts daily and sampling them for chemical analysis. Feeders were managed to ensure no more than 10% of feed per animal remained 1 h before feeding, and if feeders were empty, 10% of the feed was added to the previous day’s amount. Data on pregnancy, lambing, abortion, and mortality were recorded. Ewes had a 1-week adaptation period to the pens and diets. Ewes and lambs were weighed, and intake was measured, with weights recorded biweekly before morning feeding. Lamb intake was determined by partitioning a section of the pen or area with a gate or openings that allowed lambs to pass but not ewes. The average daily gain (ADG) of lambs before weaning was calculated by dividing the change in their body weights by the time period (15 days) between weighings. Both ewes and lambs had ad libitum access to their diets and fresh water throughout the experiment.

Various rates, including the pregnancy rate, lambing rate, abortion rate, and mortality rate, were calculated. The pregnancy rate was determined as the number of ewes that gave birth per number of ewes mated. The lambing rate was calculated as the number of lambs born per number of ewes mated. The abortion rate was determined as the number of ewes that experienced abortion per number of ewes mated, and the mortality rate was calculated as the number of lamb mortalities that occurred before weaning per number of lambs born.

Two colostrum samples, approximately 50 mL from each half of the udder, were manually collected into plastic bottles at two different time points: 1 h and 12 h after parturition. These samples were then stored at a temperature of −20 °C until they could be analyzed. To determine colostrum production, ewes were milked 1 h after giving birth. Milk production measurements were taken during the second week of lactation and continued biweekly thereafter. The lambs were separated from their mothers 12 h before milking. Milk production over a 12-h period was estimated biweekly at 08:00 AM through hand milking. Subsequently, milk production was calculated over a 24-h period, as outlined in the study by [18]. For the analysis of the chemical composition of the milk, a 125 mL sample was collected biweekly from each ewe and stored at −20 °C for later analysis.

Laboratory procedures involved processing composited diets and orts samples. These samples were dried at a temperature of 55 °C in a forced air oven until they reached a constant weight, then air equilibrated, and finally ground to pass through a 1 mm screen (Barbender Ohg, type 880845, Duisburg, Germany). These ground samples were retained for further analysis, including determining dry matter (DM) using a method involving heating at 100 °C in a forced air oven for 24 h (method 967.03) and nitrogen (N) through the Kjeldahl procedure (method 976.06) according to AOAC [19] guidelines. Furthermore, all samples underwent analysis for neutral detergent fiber (NDF) and acid detergent fiber (ADF) based on the procedures outlined by [20] with modifications suited for the Ankom2000 fiber analyzer apparatus (Ankom Technology Corporation, Macedon, NY, USA). The neutral detergent fiber analysis included the use of sodium sulfite and a heat-stable alpha-amylase. Both NDF and ADF values were reported with residual ash content.

### 2.5. Colostrum and Milk Analysis

In the analysis of colostrum and milk, colostrum samples were diluted by either one-half or one-third, depending on their viscosity, with distilled water. After reaching room temperature, these samples were analyzed for their fat and protein concentrations. Further analyses included determining solids-non-fat (SNF) content, fat content, and crude protein (CP) content. SNF content was calculated using the lactometer method. Crude protein, calculated as N × 6.38, was determined using the Kjeldahl procedure (method 976.06) [19]. Fat content was assessed according to the Gerber method (Gerber Instruments, K. Schnider and Co., AG; 8307 Langhag, Effretikon, Switzerland). The concentration of colostrum IgG was measured as a representation of the immunoglobulin content in the colostrum, using the Brix refractometer method as described by Quigley et al. [21]

Milk samples were thawed and combined for each ewe, then warmed to room temperature for analysis of their SNF, fat, and protein concentrations using an infrared milk analyzer (GNC Milking Systems, Konya, Türkiye). Additionally, the milk fatty acid profile was examined. Fat was extracted from the milk samples and subjected to transesterification into fatty acid methyl esters by methylation at room temperature with NaOH (2 M) in methanol, following the procedure described by Palmquist and Jenkins [22]. The fatty acid profile was then analyzed by gas chromatography (GC) using the Shimadzu 2010 method with a flame ionization detector (Kyoto, Japan). Milk fatty acid compositions were assessed by GC according to [20] using a Supelcowaxs-10 fused silica capillary column (60 m × 0.75 mm i.d., phase thickness 1.0 mm; Supelco Inc., Bellefonte, PA, USA). Nitrogen was used as the carrier gas, and the initial oven temperature was programmed from 60 to 70 °C at a rate of 2 °C/min, followed by an increase from 70 to 230 °C at a rate of 20 °C/min. Injector and detector temperatures were maintained at 250 °C, and the nitrogen flow rate was set at 1.2 mL/min, in line with the procedure described by Sampelayo et al. [23].

### 2.6. Statistical Analysis

IBM SPSS statistical package version 23.0 (IBM Corp., Armonk, NY, USA, Version 23.0) was used for data analysis [24]. Significance was considered at *p*-values ≤ 0.05, and values between 0.05 and 0.10 were considered tendencies. The least-squares difference (LSD) was employed to identify differences among groups. ANOVA was used to analyze differences between treatment groups concerning initial body weight. ANCOVA with initial BW as a covariate was used due to the significant random effect of initial body weight on final BW, WG, and ADG. Pregnancy, number of lambs born, number of ewes lambed, number of lambs weaned, abortions, and mortality rates were analyzed using the chi-square test, with Fisher’s exact test applied when cell counts were less than 5.

## 3. Results

### 3.1. Effects of Feeding Omega-3 Fatty Acids during Pregnancy on Ewes and Lamb Performance

The gestation length (days) is shown in Table 3. Awassi ewes fed FO and POFO had significantly longer gestation periods (*p* < 0.001) compared to other treatment groups. The initial and final body weights (BW) of ewes did not differ significantly (*p* > 0.05) among the treatments. Dry matter intake and body weight gain (BWG) were similar across all treatments. The impact of feeding omega-3 fatty acids during gestation on ewes and their lamb performance during the lactation period is presented in Table 3. Birth weight was higher (*p* < 0.001) for the FO, POFO, and FOPO groups compared to PO, with no significant difference between PO and FOPO. There were no significant differences (*p* > 0.05) in intake during the nursing period, ewes’ intake during lactation, and ewes’ body weight change during lactation among the treatments. The results regarding the number of lambs, ewes lambed, lambs weaned, abortions, and mortality are given in Table 3. There were no significant differences (*p* > 0.05) in the number of lambs at lambing, ewes lambed, lambs weaned, abortions, and mortality among the treatments according to chi-square and Fisher’s exact tests (Table 3). However, supplementing ewes with FO during late pregnancy numerically decreased the ewes’ abortion rate and the mortality of their lambs.

The results of lamb ADG before weaning are given in Table 4. Lamb ADG was significantly (*p* < 0.001) greater in the first 15 days for PO and FOPO compared to FO and POFO. From 15 days to 30 days, there were no significant differences (*p* > 0.05) in lamb ADG. From 30 days to 60 days, ADG was significantly greater (*p* < 0.001) for FO and POFO compared to PO and FOPO. On average, there were no significant differences (*p* > 0.05) in lamb ADG.

### 3.2. Effect on Colostrum and Milk Yield and Composition

Colostrum yield, fat content, and protein content were significantly higher (*p* < 0.001) in ewes fed PO and FOPO compared to those fed FO and POFO diets. However, immunoglobulin G (IgG) content was significantly lower (*p* < 0.001) for ewes fed PO and FOPO compared to those fed POFO and FO diets (Table 5). The results of milk fat, SNF, and protein are shown in Table 6. Milk fat, SNF, and protein percentages were significantly higher (*p* < 0.001) for PO and FOPO compared to FO and POFO during the first 15 days of lactation, but there was no significant effect during the rest of the study. Milk yield during the lactation period is presented in Table 7. Awassi ewes fed PO and FOPO had significantly greater (*p* < 0.001) milk production than other treatment groups during the first 30 days of lactation, with no differences between groups after 30 days of lactation. On average, Awassi ewes fed PO and FOPO had significantly greater (*p* < 0.001) milk production than other treatment groups. Fish oil supplementation to ewes reduced (*p* < 0.001) the concentrations of C16:0, C18:0, and C18:1 cis-9 in milk fat (Table 8). In contrast, concentrations of C18:1 trans, conjugated linoleic acid (CLA, cis-9, trans-11), C18:3 (n-3), C20:5 (n-3), and C22:6 (n-3) were increased (*p* < 0.001), with no effect on C18:2 n-6 and C20:4 n-6 in the milk fatty acid profile due to fish oil supplementation. There was no effect of fish oil supplementation on the ewes’ milk fatty acid profile in the 90 days after lambing, as the ewes were offered the same diet without fish oil during this period.

## 4. Discussion

### 4.1. Effects of Feeding Omega-3 Fatty Acids during Pregnancy on Ewes and Lamb Performance

In agreement with our results, the inclusions of PUFA n-3 in sheep [25] led to an extension of gestation length and are thought to be associated with a reduction in the synthesis of two series prostaglandins, due to increased EPA concentrations [12,26]. Capper et al. [11] reported that ewes fed a diet containing FO during the third trimester of gestation increased gestation length by two days compared with palm oil. The AA and EPA act as major prostaglandin (PG) precursors within the ruminant; the bioactive dienoic PGE2 and PGF2α are synthesized from AA, whereas PGE3 is produced from EPA. When an increase in the ratio of EPA to AA available to the ewe results in a shift in PG production from bioactive dienoic PGs, which have an established role in the induction of parturition, toward less active trienoic PG [27], this is thought to be associated with a reduction in uterine muscle contractility due to an increased supply of DHA [28]. In another study, [13] demonstrated a delay in the initiation of glucocorticoid-induced parturition in ewes infused with FO, with concurrent reductions in maternal estradiol and prostaglandin-H-synthetase-2. Concurrent with the results obtained herein, [29] also reported that supplementing ewes with FO during late pregnancy and lactation did not affect DM intake and the dietary fat source did not affect the daily intake of pregnant and lactating ewes. Although many studies have reported that the inclusion of FO in ruminant rations led to a reduction in DM intake [30,31], such an effect was not observed in the current study. However, the previous studies did not include a control fat source in the control diet; therefore, the observed results may have resulted from similar effects of FO and PO upon DM intake. The depressive effect of PUFAs upon DM intake has been attributed to the toxic effects of PUFAs on rumen microbes, although the use of protected FOs appears to negate these effects [32]. Therefore, PUFA supplementation may have metabolic effects upon DM intake via shifts in ruminal biohydrogenation leading to an increased supply of USFAs at the duodenum [33]. In agreement with our results, [34] reported that supplementing ewes with FO during late pregnancy increased lamb birth weight. The increase in the birth weight of lambs may be attributed to the long gestation period, as discussed earlier in this study. A relationship between fetal growth rates and gestation length existed, with the fetal growth rates during the late stage of pregnancy being 0.15–0.2 kg/day [11,25]. This increase in gestation length may have resulted in a physiologically higher lamb birth weight at parturition [13,34].

The increase in gestation length may be associated with an increase in the birth weight of lambs born from ewes supplemented with FO and thus negatively correlated with mortality rates [11,25,35]. Moreover, weak lambs with low birth weight were unable to suckle a sufficient amount of colostrum, and the immunoglobulin level in their serum was low. Physical weakness and low immunoglobulin lead to increased mortality in lambs with low birth weight [36]. In our results, groups that received FO in their diets in the late pregnancy period had a lower mortality rate (numerically), and thus increasing lamb birth weight is negatively correlated with mortality rate; this agrees with the results of many studies that supplemented ewes with FO in late pregnancy [37]. A diet high in omega-3 may improve embryo survival by reducing PGF2α synthesis, which may prevent regression of the CL, allowing continued secretion of P4 [5]. Caldari-Torres et al. [6] reported a positive effect of n-3 on embryo survival. In our study, the abortion rate decreased numerically in ewes fed FO in the late gestation period without influencing the lambing rate, and the lamb mortality rate before weaning. In the late pregnancy and lactation periods, the FO supplementation for ewes led to a reduced lamb growth rate by reduced milk yield and composition [29,38]. Milk is the only nutrient source available to sucking lambs within the first period of life and milk yield and composition are reduced by the effect of FO in an ewe’s diet. Therefore, the lamb growth rate was also reduced [29]. However, in our study in the first 30 days, the lamb ADG from ewes fed FO in the late pregnancy period was less than the lamb ADG for ewes fed PO in the late pregnancy period, but on average there was no difference between groups in ADG. The reduction in ADG for lambs in the first 30 days may be related to the reduction in milk production and composition in the first 30 days of the lactation period; after that, the milk production and composition were the same for all groups, and then the lambs returned to the normal gain.

### 4.2. Effect on Colostrum and Milk Yield and Composition

The colostrum yield, fat, and protein contents were lower in FO diets at the late period of gestation compared with FO-free diets during the late period of gestation. Conversely, IgG was lower in the FO-free diet during the late period of gestation. The nutritional status of ewes during late gestation plays a significant role in the quality and quantity of colostrum produced. Adequate nutrition during this period is essential for the development of the mammary glands and the synthesis of colostral components [2,29]. Also, PUFAs (especially EPA and DHA) from fish oil diets can stimulate IgG synthesis [39]. In agreement with our results, [31,40] it was found that colostrum yield, fat, and protein decreased following n-3 supplementation with FO [11]. The most widely accepted hypothesis for the cause of diet-induced milk fat depression in dairy ruminants involves direct inhibition of lipid synthesis in the mammary gland, in response to FA intermediates (trans-C18:1 and related metabolites, such as conjugated linoleic acid, CLA), formed during partial biohydrogenation of USFAs in the rumen [41]. In this study, the inclusion of FO in the diet of ewes, especially in late gestation, reduced milk yield. This effect was seen only in the first 30 days of the lactation period and after 30 days of the milking period; all groups had similar milk yield, and the reduction in milk production and changes in its composition were observed over a short timescale, associated with the presence of FO in the diet. Reduced milk yield and changes in its composition have been observed for dairy cattle supplemented with FO [38]. The reduction in milk yield could be attributed to the reduction in the DM intake in cattle and ewes [30,31,40]. In the current study, we did not have a significant difference in DMI between treatments in the pregnancy and lactation period. Capper et al. [11] supplemented FO to pregnant ewes at the late gestation period and demonstrated a reduction in milk yield and milk composition. The negative effect of FO on milk production was attributed to hydrogenation intermediaries, including trans-10 18:1, trans-10, and cis-12 CLA [42]. As expected, the inclusion of FO in pregnant ewes in the late pregnancy period led to an increase in C18:1 trans, CLA (cis-9, trans-11), C18:3 (n-3), EPA C20:5 (n-3), and DHA C22:6 (n-3) and a decrease in C16:0, C18:0, and C18:1 cis-9, without effect in C18:2 n-6 and C20:4 n-6. In agreement with previous results in dairy cattle, Shingfield et al. [35] reported that the inoculation of FO in dairy cattle led to an increasing amount of 20:5n-3 and 22:6n-3 in milk.

Many studies have reported that the supplementation of rumen-protected marine oil, which is rich in EPA and DHA, was able to noticeably increase the concentration of these LCFA in milk fat compared with that of milk from untreated sheep [43,44]. Nevertheless, the transfer of EPA and DHA from the diet into milk was markedly higher than that reported for dairy cows [45] and dairy goats [44]. Another study reported that 20:5n-3 and 22:6n-3 exceeded 0.2 g/100 g of milk FA when FO was added to the diet at 2% of DM [46]. In agreement with the results observed by Capper et al. [29], the current study showed that the concentration of C16:0 within milk fat appeared to be depressed in ewes offered FO. However, rather than a depressive effect of PUFA supplementation per se, this may be attributed to the high dietary concentration of this FA in the diets that have PO in late pregnancy. The transfer of EPA and DHA to milk is in agreement with Capper et al. [29]. It was reported that EPA and DHA were transferred into milk at rates between 6.8% and 8.1% of dietary intake [47], and, also, there are references that transfer 10% for EPA and 17% for DHA in ewes fed FO [29]. In the present study, the increase in CLA (cis-9, trans-11) led to an increase in 18:1 trans in milk fat, induced by 18:1 trans which is mainly endogenously produced by the mammary desaturation of 18:1 trans by stearoyl-CoA desaturase enzyme [41], and by inhibition of 18:1 trans biohydrogenation in the rumen [38]. In the present study, there was no effect of FO supplementation of ewes in the 90 days after lambing on the milk FA profile. The reduction in milk production and composition was over a short timescale, associated with the presence of FO in the diet.

## 5. Conclusions

Supplementing the diets of pregnant Awassi ewes with fish oil during pregnancy resulted in longer gestation periods and increased lamb birth weight, as well as improved IgG content in colostrum. Therefore, lamb growth or survival would be improved in this context. However, it had negative effects on colostrum yield, fat, and protein content. Fish oil supplementation also influenced milk production and composition, with a temporary reduction in milk yield and changes in fatty acid profiles. This suggests that fish oil can be used to enhance milk fat content, particularly polyunsaturated fatty acids (PUFAs) such as DHA, EPA, and CLA. These fatty acids are beneficial for human health. Overall, including fish oil in the diets of pregnant Awassi ewes can be a valuable intervention to improve lamb birth weight and milk fat quality, despite some temporary effects on colostrum and milk composition. This approach may enhance production profitability in arid and semi-arid regions.

## Figures and Tables

**Table 1:** TMR content and nutrient composition used in early and late gestation.

	Early Gestation			
Ingredients (% DM)	PO	POFO	FOPO	FO
Barley grain	23.40	23.40	23.40	23.40
Soybean meal, 44% CP (solvent ext.)	6.77	6.77	6.77	6.77
Wheat straw	54.09	54.09	54.09	54.09
Wheat bran	11.56	11.56	11.56	11.56
Fish oil (FO)	-	-	2.40	2.40
Palm oil (PO)	2.40	2.40	-	-
Salt	0.38	0.38	0.38	0.38
Limestone	1.00	1.00	1.00	1.00
Mineral vitamin premix ^a^	0.40	0.40	0.40	0.40
Nutrient contents				
DM%	91.31	90.62	90.59	89.69
CP%	10.18	9.52	9.84	9.44
ADF%	27.46	27.36	28.16	27.86
NDF%	48.14	48.51	48.36	49.12
EE%	4.17	4.22	4.36	4.18
ME (Mcal/Kg) ^b^	2.43	2.61	2.36	2.33
	Late Gestation			
	PO	POFO	FOPO	FO
Barley grain	26	26	26	26
Soybean meal, 44% CP (solvent ext.)	6.5	6.5	6.5	6.5
Wheat straw	53.25	53.25	53.25	53.25
Wheat bran	10.85	10.85	10.85	10.85
Fish oil (FO)	-	2.1	-	2.1
Palm oil (PO)	2.1	-	2.1	0
Salt	0.5	0.5	0.5	0.5
Limestone	0.5	0.5	0.5	0.5
Mineral vitamin premix	0.3	0.3	0.3	0.3
Nutrient contents				
DM%	90.57	89.67	90.12	90.54
CP%	10.62	10.54	11.02	10.41
ADF%	26.62	27.12	26.51	26.86
NDF%	48.42	49.10	48.35	48.80
EE%	4.34	4.14	4.25	4.17
ME (Mcal/Kg) ^b^	2.65	2.24	2.52	2.31

^a^ Composition per kg: vitamin A, 2,000,000 IU; vitamin D3, 40,000 IU; vitamin E, 400 mg; Mn, 12.80 g; Zn, 9.00 g; I, 1.56 g; Fe, 6.42 g; Cu, 1.60 g; Co, 50 mg; Se, 32 mg. ^b^ Calculated based on tabular values using NRC [17].

**Table 2:** Fatty acid profile of fish oil and palm oil.

Fatty Acid	Fish Oil	Palm Oil
Lauric (C12:0)	-	0.17
Myristic (C14:0)	6.2	0.94
Pentadecanoic acid (C15:0)	0.72	-
Palmitic (C16:0)	20.4	41.98
Palmitoleic (C16:1)	8.15	0.18
Heptadecanoic (C17:0)	1.1	0.08
Heptadecenoic (C17:1)	0.56	0.03
Stearic (C18:0)	5.4	2.2
Oleic (C18:1)	16.1	41.6
Linoleic (C18:2)	2.11	10.9
Linolenic (C18:3)	0.63	0.16
Arachidic (C20:0)	0.4	0.33
Gadoleic (C20:1)	0.8	0.12
Behenic (C22:0)	-	0.06
Lignoceric (C24:0)	-	0.06
Arachidonic acid (ARA, C20:4)	2.7	-
Eicosapentaenoic acid (EPA, C20:5)	10.1	-
Dicosapentaenoic acid (DPA, C22:5)	3.1	-
Dicosahexaenoic acid (DHA, C22:6)	16.3	-

**Table 3:** Effect of feeding fish oil during gestation on maternal and lamb performance.

	Treatments ^1^					
	PO	POFO	FOPO	FO	SEM ^2^	*p*-Value
Gestation length, day	145 ^a^	147 ^b^	146 ^a^	149 ^b^	0.48	<0.001
Dry matter intake, g/day	1294	1282	1291	1287	10.08	0.952
Initial body weight, kg	55.4	58.0	58.0	56.5	1.77	0.523
Final body weight, kg	70.0	72.1	73.0	71.1	1.46	0.432
Ewe BWG, kg	14.5	14.2	15.1	14.6	1.29	0.734
Birth weight (kg)	4.3 ^a^	4.9 ^b^	4.8 ^ab^	5.0 ^b^	0.10	0.049
Lamb intake (g/day)	93.5	93.5	93.5	93.4	0.48	1.000
Ewes DMI during lactation (g/day)	1802	1801	1802	1819	3.38	0.232
Ewes BWC ^3^ during lactation (kg)	−2.8	−2.8	−2.9	−2.5	0.09	0.601
Number of lambs	18	19	18	19		0.054 *
Ewes lambed	14	16	15	16		0.180 *
Lambs weaned	15	18	16	19		0.588 *
Abortion	1	0	1	0		1.000 **
Mortality	3	1	2	0		1.000 **

^1^ Treatment diets were as follows: (1) diet containing PO from 0 to 150 days of pregnancy (PO; n = 15), (2) diet containing PO from 0 to 65 days of pregnancy, then diet containing FO 66—150 days of pregnancy (POFO; n = 16), (3) diet containing FO from 0 to 65 days of pregnancy, then diet containing PO from 66 to 150 days of pregnancy (FOPO; n = 16), and (4) diet containing FO from 0 to 150 days of pregnancy (FO; n = 16). ^2^ SEM: Standard error of pooled means. ^3^ BWC: Body weight change. ^ab^ Means within a row with different superscripts differ (*p* < 0.05). * Chi-square test. ** Fisher’s exact test for cell counts less than 5.

**Table 4:** Effect of fish oil during pregnancy on lamb average daily gain before weaning.

	Treatments ^1^					
Period (Day)	PO	POFO	FOPO	FO	SEM ^2^	*p*-Value
1–15 (g)	247 ^a^	204 ^b^	258 ^a^	183 ^b^	0.006	<0.001
15–30 (g)	245	247	253	221	0.005	0.150
30–45 (g)	241 ^a^	287 ^b^	247 ^a^	269 ^ab^	0.006	0.034
45–60 (g)	243 ^a^	288 ^b^	242 ^a^	272 ^ab^	0.006	0.015
1–60 (g)	244	256	250	236	0.005	0.531

^1^ Treatment diets were as follows: (1) diet containing PO from 0 to 150 days of pregnancy (PO; n = 15), (2) diet containing PO from 0 to 65 days of pregnancy, then diet containing FO 66—150 days of pregnancy (POFO; n = 16), (3) diet containing FO from 0 to 65 days of pregnancy, then diet containing PO from 66 to 150 days of pregnancy (FOPO; n = 16), and (4) diet containing FO from 0 to 150 days of pregnancy (FO; n = 16). ^2^ SEM: Standard error of pooled means. ^ab^ Means within a row with different superscripts differ (*p* < 0.05).

**Table 5:** Effect of feeding fish oil during gestation on colostrum parameters.

	Treatments ^1^					
	PO	POFO	FOPO	FO	SEM ^2^	*p*-Value
Colostrum yield (kg/d)	1.6 ^a^	1.3 ^b^	1.5 ^a^	1.2 ^b^	1.45	<0.001
Fat %	10.1 ^a^	8.2 ^b^	10.2 ^a^	8.1 ^b^	0.15	<0.001
Protein %	13.0 ^a^	11.6 ^b^	13.0 ^a^	11.4 ^b^	0.06	<0.001
IgG (g/L)	59.2 ^b^	77.2 ^a^	60.6 ^b^	78.5 ^a^	1.24	<0.001

^1^ Treatment diets were as follows: (1) diet containing PO from 0 to 150 days of pregnancy (PO; n = 15), (2) diet containing PO from 0 to 65 days of pregnancy, then diet containing FO 66–150 days of pregnancy (POFO; n = 16), (3) diet containing FO from 0 to 65 days of pregnancy, then diet containing PO from 66 to 150 days of pregnancy (FOPO; n = 16), and (4) diet containing FO from 0 to 150 days of pregnancy (FO; n = 16). ^2^ SEM: Standard error of pooled means. ^ab^ Means within a row with different superscripts differ (*p* < 0.05).

**Table 6:** Effect of fish oil during pregnancy on milk fat, SNF, and protein composition.

	Treatments ^1^					
	PO	POFO	FOPO	FO	SEM ^2^	*p*-Value
Fat %						
15 days	7.42 ^a^	6.37 ^b^	7.26 ^a^	6.29 ^b^	0.10	<0.001
45 days	6.54	6.45	6.70	6.48	0.08	0.715
90 days	6.16	6.02	6.17	6.31	0.10	0.781
SNF %						
15 days	11.43 ^a^	10.68 ^b^	11.15 ^a^	10.42 ^b^	0.08	<0.001
45 days	10.80	10.51	10.66	10.58	0.06	0.387
90 days	10.62	10.35	10.41	10.32	0.06	0.251
Protein %						
15 days	4.22 ^a^	3.82 ^b^	4.36 ^a^	3.66 ^b^	0.05	<0.001
45 days	3.80	3.75	3.69	3.72	0.03	0.661
90 days	3.40	3.55	3.69	3.46	0.04	0.071

^1^ Treatment diets were as follows: (1) diet containing PO from 0 to 150 days of pregnancy (PO; n = 15), (2) diet containing PO from 0 to 65 days of pregnancy, then diet containing FO 66–150 days of pregnancy (POFO; n = 16), (3) diet containing FO from 0 to 65 days of pregnancy, then diet containing PO from 66 to 150 days of pregnancy (FOPO; n = 16), and (4) diet containing FO from 0 to 150 days of pregnancy (FO; n = 16). ^2^ SEM: Standard error of pooled means. ^ab^ Means within a row with different superscripts differ (*p* < 0.05)

**Table 7:** Effect of fish oil during pregnancy on milk yield (kg) during the lactation period.

	Treatments ^1^					
Lactation Days	PO	POFO	FOPO	FO	SEM ^2^	*p*-Value
15	1.70 ^a^	1.32 ^b^	1.69 ^a^	1.27 ^b^	0.03	<0.001
30	1.81 ^a^	1.39 ^b^	1.83 ^a^	1.38 ^b^	0.03	<0.001
45	1.62	1.51	1.58	1.52	0.02	0.122
60	1.46	1.42	1.48	1.40	0.02	0.524
75	1.36	1.36	1.39	1.32	0.02	0.731
90	1.33	1.30	1.33	1.27	0.02	0.810
Average	1.54 ^a^	1.38 ^b^	1.55 ^a^	1.36 ^b^	0.03	<0.001

^1^ Treatment diets were as follows: (1) diet containing PO from 0 to 150 days of pregnancy (PO; n = 15), (2) diet containing PO from 0 to 65 days of pregnancy, then diet containing FO 66–150 days of pregnancy (POFO; n = 16), (3) diet containing FO from 0 to 65 days of pregnancy, then diet containing PO from 66 to 150 days of pregnancy (FOPO; n = 16), and (4) diet containing FO from 0 to 150 days of pregnancy (FO; n = 16). ^2^ SEM: Standard error of pooled means. ^ab^ Means within a row with different superscripts differ (*p* < 0.05).

**Table 8:** Effect of fish oil during pregnancy on milk fatty acid composition.

	Treatments ^1^					
Fatty Acid (g/100 g Fatty Acid)	PO	POFO	FOPO	FO	SEM ^2^	*p*-Value
Day 14						
C16:0	25.2 ^a^	20.4 ^b^	25.6 ^a^	20.6 ^b^	0.52	<0.001
C18:0	13.8 ^a^	8.0 ^b^	13.7 ^a^	8.00 ^b^	0.60	<0.001
C18:1 trans	3.7 ^a^	5.5 ^b^	3.6 ^a^	5.5 ^b^	0.21	<0.001
C18:1 cis-9	26.7 ^a^	22.1 ^b^	26.8 ^a^	21.9 ^b^	0.50	<0.001
C18:2 n-6	1.8	1.6	1.7	1.5	0.05	0.089
CLA(cis-9, trans11)	1.02 ^a^	1.45 ^b^	0.96 ^a^	1.50 ^b^	0.06	<0.001
C18:3 n-3	0.37 ^a^	0.61 ^b^	0.36 ^a^	0.60 ^b^	0.03	<0.001
C20:4 n-6	0.01	0.19	0.10	0.18	0.05	0.123
C20:5 n-3 EPA	0.03 ^a^	0.61 ^b^	0.04 ^a^	0.60 ^b^	0.06	<0.001
C22:6 n-3 DHA	0.02 ^a^	0.69 ^b^	0.03 ^a^	0.66 ^b^	0.07	<0.001
Day 90						
C16:0	24.7	24.8	24.9	24.7	0.08	0.798
C18:0	13.3	13.1	13.0	12.8	0.07	0.103
C18:1 trans	3.25	3.05	3.25	3.05	0.05	0.368
C18:1 cis-9	25.9	25.7	25.7	25.7	0.11	0.897
C18:2 n-6	1.4	1.4	1.4	1.3	0.04	0.603
CLA (cis-9, trans-11)	0.96	0.95	0.95	0.96	0.006	0.990
C18:3 n-3	0.32	0.30	0.30	0.29	0.006	0.350
C20:4 n-6	0.08	0.08	0.07	0.09	0.003	0.084
C20:5 n-3	0.03	0.09	0.03	0.02	0.001	0.476
C22:6 n-3	0.02	0.02	0.02	0.01	0.002	0.660

^1^ Treatment diets were as follows: (1) diet containing PO from 0 to 150 days of pregnancy (PO; n = 15), (2) diet containing PO from 0 to 65 days of pregnancy, then diet containing FO 66–150 days of pregnancy (POFO; n = 16), (3) diet containing FO from 0 to 65 days of pregnancy, then diet containing PO from 66 to 150 days of pregnancy (FOPO; n = 16), and (4) diet containing FO from 0 to 150 days of pregnancy (FO; n = 16). ^2^ SEM: Standard error of pooled means. ^ab^ Means within a row with different superscripts differ (*p* < 0.05).

## Data Availability

Research data will be provided upon request.
